# Efficiency and Safety of Repeated Vertebroplasty for Adjacent Segment Fractures

**DOI:** 10.3390/jcm14010166

**Published:** 2024-12-31

**Authors:** Bo-Sheng Wu, Ming-Cheng Hsu, Yu-Cheng Yao, Hsi-Hsien Lin, Po-Hsin Chou, Shih-Tien Wang, Ming-Chao Chang, Wei Hsiung, Chien-Yuan Wang, Kuan-Jung Chen

**Affiliations:** 1 Department of Medical Education, Kaohsiung Chang Gung Memorial Hospital, Kaohsiung 83301, Taiwan; boshengw16507@gmail.com; 2 Department of Medical Education, National Cheng Kung University Hospital, No.138, Sheng Li Road, North Dist., Tainan 11558, Taiwan; n172102@mail.hosp.ncku.edu.tw; 3 Department of Orthopedics, Taipei Veterans General Hospital, Taipei 11217, Taiwan; orthycyao@gmail.com (Y.-C.Y.); hsihsienlin@gmail.com (H.-H.L.); choupohsin@gmail.com (P.-H.C.); stwang@vghtpe.gov.tw (S.-T.W.); mcchang@vghtpe.gov.tw (M.-C.C.); 4 Department of Orthopedics, School of Medicine, National Yang Ming Chiao Tung University, Taipei 11221, Taiwan; edward.kuma@gmail.com; 5 Ministry of Health and Welfare, Kinmen Hospital, Kinmen County 89142, Taiwan; 6 Department of Orthopedics, Shin Kong Wu Ho-Su Memorial Hospital, Taipei 11101, Taiwan; 7 Department of Orthopedics, China Medical University Hsinchu Hospital, Hsinchu County 30058, Taiwan; orthodoc66@gmail.com; 8 Department of Orthopedics, College of Medicine, China Medical University, Taichung 40402, Taiwan

**Keywords:** adjacent vertebral fracture, spinal fracture, vertebroplasty, cement augmentation, complication

## Abstract

**Objectives:** To review the outcomes of patients who underwent repeated vertebroplasty (VP) surgery for adjacent segment fractures (ASF), defined as new osteoporotic vertebral fractures occurring at levels immediately above or below a previously treated vertebra. **Methods:** From 1 January 2018, to 31 December 2020, forty-one patients who developed ASF following initial VP and underwent repeated VP were enrolled in our study. Radiographic measurements included single and two-segment kyphotic angles (SKA and TKA), and anterior and mid-vertebral body height (AVH and MVH). Patient-reported outcomes included back pain assessed with the visual analog scale (VAS) and the Oswestry Disability Index (ODI). **Results:** The procedure significantly reduced the mean single kyphotic angle (SKA) by 4.8° ± 6.8° (*p* < 0.01) and the two-segment kyphotic angle (TKA) by 3.0° ± 7.9° (*p* = 0.02), along with increases in anterior and mid-body height by 0.3 ± 0.5 cm and 0.3 ± 0.6 cm (both *p* < 0.01). However, there was a slight restoration loss in SKA and TKA at a 20.1-month follow-up. Patient-reported outcomes revealed substantial pain reduction, with the VAS score dropping from 8 to 1 (*p* < 0.0001) and the mean ODI score improving from 59.7 to 28.9 (*p* < 0.0001). The complication rate was 34.1%, including nonunion, de novo fractures, cement leakage, and neurological deficits. Additionally, 7.3% of cases necessitated further surgical interventions. **Conclusions:** Repeated VP for ASF improves vertebral alignment parameters and patient-reported outcomes, but with a high rate of complications and reoperation.

## 1. Introduction

Osteoporotic vertebral compression fractures (OVCFs) are the most common fractures observed in individuals with osteoporosis. The prevalence of OVCFs in individuals aged 65 years and above ranges from 10% to 20% in men and 18% to 30% in women [1]. The incidence escalates with advancing age [2]. If left untreated, vertebral fractures can result in changes to the biomechanics of the spine, increasing the risk of additional vertebral fractures and negatively impacting the patient’s quality of life [3,4,5]. Conservative treatments are typically recommended for initial management, but patients who fail to respond may require surgical intervention. However, conventional open instrumentation can lead to surgical complications and may be challenging in elderly patients with medical comorbidities and fragile bones [6]. Vertebral augmentation procedures such as vertebroplasty (VP), on the contrary, are a minimally invasive and effective treatment option for pain relief and stabilization of the vertebra and are the treatment of choice for many patients with OVCFs [7,8,9]. The advantages of VP include its minimally invasive nature, fast recovery time, and a high success rate in terms of improving patient-reported outcomes [10,11,12].

On the other hand, adjacent segment fractures (ASF) are a common complication following VP. The majority of ASF occurred within 2 months after the procedure. The reported occurrence rates of ASF within 24 months of VP range from 18.9% to 26.0% [6,13,14,15], highlighting the significance of this issue. ASF can lead to further spinal instability, pain, neurostenosis, and decreased quality of life for patients [6,16]. Despite its clinical importance, the management of ASF remains challenging, as no standardized treatment approach has been established. While several treatment options exist for ASF, repeated VP has emerged as a preferred approach in clinical practice [16,17]. This preference is supported by several factors: the presence of previous cement augmentation and potentially compromised bone quality in adjacent segments makes open surgical approaches particularly challenging, elderly patients who develop ASF often have the same contraindications to open surgery that led to choosing VP for their initial fracture, and conservative treatment may be insufficient due to the biomechanical alterations caused by the previous VP.

However, the decision to perform repeated VP for ASF requires careful consideration, as surgeons must weigh the minimally invasive benefits against potential risks. While existing literature supports the use of repeated VP, systematic outcome data, particularly regarding complications and reoperation rates, remain limited. Current evidence gaps include detailed analysis of radiographic outcomes, patient-reported improvements, and procedure-related complications. Therefore, this study aims to evaluate the clinical outcomes, radiographic parameters, and complications in patients who underwent repeated VP for ASFs.

## 2. Materials and Methods

### 2.1. Study Design

**Figure 1:**
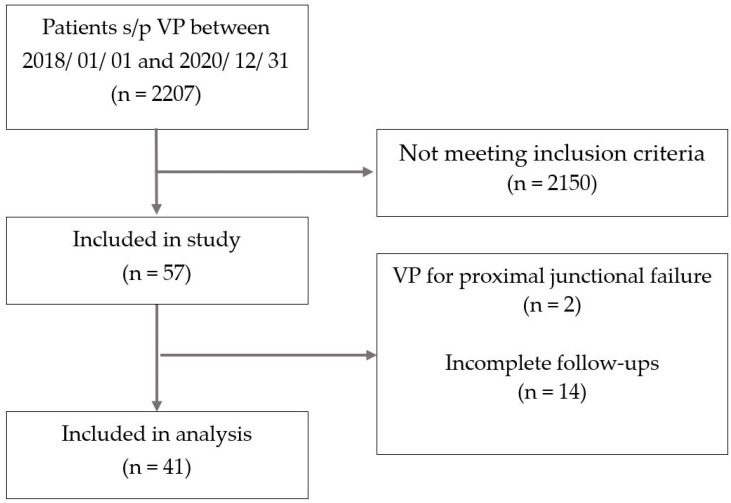
Study flowchart.

After obtaining institutional review board approval, we conducted a retrospective chart and radiographic review of the 2207 patients who received VP at Taipei Veterans General Hospital between 1 January 2018, and 31 December 2020. The inclusion criteria were a diagnosis of ASF and receiving repeated VP for ASF after the initial VP. Exclusion criteria consisted of spinal malignancy or infection, VP for proximal junctional failure treatment, and incomplete preoperative, intraoperative, or postoperative follow-up. Of the 2207 patients who underwent VP during the study period, a total of 133 patients met our inclusion criteria for repeated procedures. Within this group, 76 patients received treatment for non-adjacent segments, and, more specifically, 57 of them underwent repeated VP to ASF. Among this subset, 14 patients had incomplete follow-up data, while an additional 2 patients were treated for malignancy-associated vertebral compression fractures and thus were excluded from our analysis (Figure 1). VP was performed according to a standardized protocol under local anesthesia. Using C-arm imaging guidance, a Jamshidi needle was percutaneously inserted into the pedicle through a 2–3 mm incision. Polymethyl-methacrylate cement was subsequently injected. Patients were discharged the same day or the following day if no postoperative acute complications or discomfort occurred. Postoperative bracing was prescribed for three months. All patients attended follow-up appointments at outpatient clinics at 1-, 4-, 8-, and 12-weeks post-surgery, followed by a 6-month visit and annual visits thereafter. This follow-up schedule was designed to align with both the natural history of ASF development (with literature showing the majority of cases occurring within 2 months post-procedure [13,14,15]) and standard post-vertebroplasty monitoring protocols. Postoperative bracing was prescribed for three months.

### 2.2. Outcome Measurements

Patient demographic and perioperative clinical characteristics, including age, sex, body mass index (BMI), comorbidities, bone mineral density, surgical level, and complications, were obtained from the medical records. The diagnosis of ASF was confirmed through thoracolumbar spine X-ray and magnetic resonance imaging (MRI), while malignancy and spinal infection were excluded based on characteristic MRI findings. In cases where imaging findings were ambiguous, histological confirmation was obtained. Cases with confirmed malignancy-associated fractures were excluded from the study. All included patients completed follow-up assessments at least 6 months postoperatively. Consecutive anteroposterior and lateral images were taken postoperatively by the same team of adequately trained radiologic technicians at each follow-up visit.

**Figure 2:**
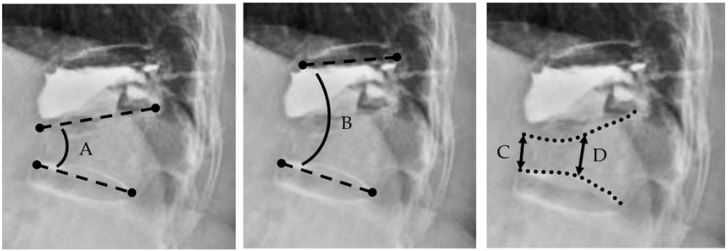
Radiographic Measurements. (A) single kyphotic angle (SKA), (B) two-segment kyphotic angle (TKA), (C) anterior vertebral body height (ABH), (D) mid-vertebral body height (MBH).

Radiographic parameters included the kyphotic angles, and the vertical height of the vertebral body was assessed on the lateral radiographs. Measurements were performed using a picture archiving and communication system (SmartIris 1.3.0.14, Taiwan Electronic Data Processing Corp., Taipei, Taiwan). The Cobb method was used to quantify the kyphotic angles [18]. The single kyphotic angle (SKA) was defined as the measurement at the newly fractured vertebra. In cases where ASF involved multiple segments, the SKA was calculated as the sum of the kyphotic angles across all affected vertebrae. The two-segment kyphotic angle (TKA) measured the overall kyphotic deformity encompassing both the previously treated vertebrae and all newly fractured adjacent segments. The anterior vertebral height (AVH) was defined as the linear distance between two points located at the anterior edge of the superior and inferior bony endplate. The middle vertebral height (MVH) was measured by connecting the midpoint of the superior and inferior vertebral endplates. All parameters were independently assessed by three authors, and the average of their measurement was recorded for each parameter. (Figure 2)

Pre- and post-operative patient-reported outcomes were assessed using the Oswestry Disability Index (ODI) questionnaire and Visual Analog Scale (VAS) for back pain intensity. ODI quantifies disability related to back pain through 10 questions, with responses presented on a 6-point scale. Scores were expressed as a percentage ranging from 0% (no disability) to 100% (completely disabled, bedridden). The VAS score ranged from 0 (no pain) to 10 (worst conceivable pain). Complication rates and subsequent reoperation rates were also reviewed from medical records.

### 2.3. Statistical Analysis

Statistical analysis was performed using MedCalc for Windows, Version 20 (MedCalc Software, Ostend, Belgium). Data normality was assessed using the Shapiro–Wilk test, and all variables were found to be normally distributed. Continuous data are presented as mean ± standard deviation, and categorical data are presented as number and percentage.

For radiographic measurements, interobserver and intraobserver reliability were evaluated using intraclass correlation coefficients (ICC) with 95% confidence intervals. ICC values were interpreted as: poor (<0.50), moderate (0.50–0.75), good (0.75–0.90), and excellent (>0.90).

Paired *t*-tests were used to compare pre- and post-operative continuous variables (kyphotic angles, vertebral heights, VAS, and ODI scores). Independent *t*-tests were used to compare continuous variables between groups. For categorical variables, chi-square tests were used for between-group comparisons. Statistical significance was set at *p* < 0.05. No data were missing from the analyzed variables.

## 3. Results

**Figure 3:**
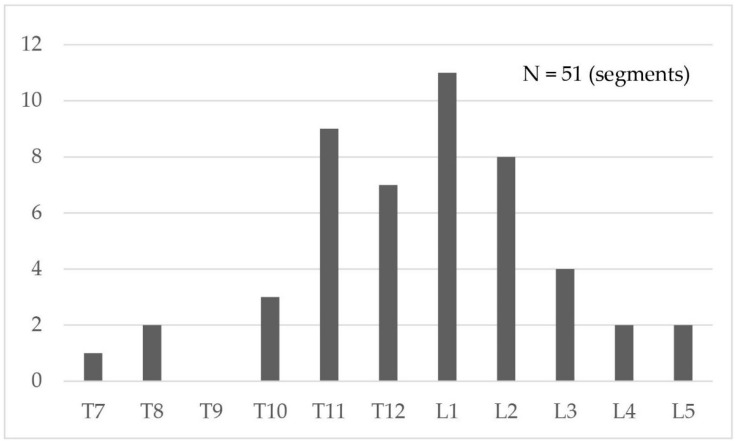
Distribution of adjacent segment fractures in vertebral levels.

A total of 41 patients with 51 affected vertebrae were included in the analysis. The study population had a mean age of 80.0 ± 7.2 years and a mean bone mineral density T-score of −2.6 ± 1.2. The majority of patients were female (37 cases). Ten patients underwent VP for multiple levels: five patients had fractures in two superior adjacent segments, four patients had fractures in two inferior adjacent segments, and one patient had concurrent superior and inferior adjacent fractures. All multiple-level VPs were performed in the same surgical session. Patient demographics and clinical characteristics are summarized in Table 1, and the distribution of affected vertebral levels is shown in Figure 3.

Radiographic measurements demonstrated excellent reliability, with ICC values of 0.93 (95% CI: 0.88–0.96) for intra-observer reliability and 0.89 (95% CI: 0.84–0.93) for inter-observer reliability. Repeated VP significantly improved spinal alignment, reducing the mean SKA by 4.8° ± 6.8° (*p* < 0.01) and TKA by 3.0° ± 7.9° (*p* = 0.02). The procedure also significantly increased both anterior and mid-body vertebral heights by 0.3 ± 0.5 cm (*p* < 0.01) and 0.3 ± 0.6 cm (*p* < 0.01), respectively (Table 2).

**Figure 4:**
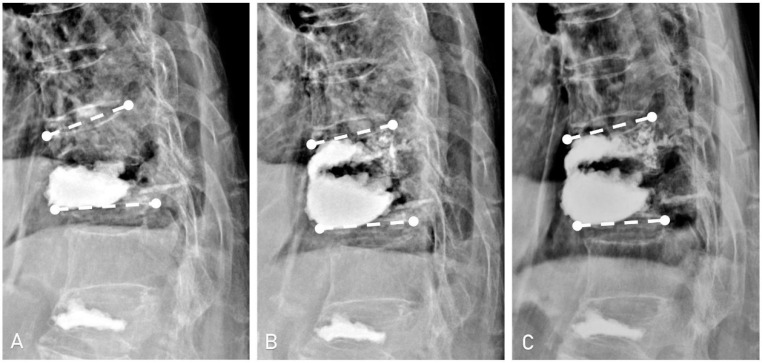
Radiographs of a 93-year-old female patient with adjacent segment fracture following vertebroplasty. Initial vertebroplasty was performed for a T12 compression fracture. (**A**): A subsequent radiograph two months later revealed an adjacent T11 vertebral fracture. (**B**): The patient underwent vertebroplasty of T11 vertebra, resulting in improved single vertebrae kyphotic angle from 21.0° to 7.1°, and an increase in anterior vertebral body height from 13.8 mm to 16.7 mm and middle vertebral height from 7.0 mm to 15.1 mm. (**C**): Follow-up at 24 weeks showed slightly increased kyphotic angle to 9.9° with reduced anterior and middle vertebral body height at 15.8 mm and 10.8 mm, respectively.

At the final radiologic follow-up (mean 20.1 ± 11.5 months), some loss of the initial correction was observed. The SKA increased an average of 1.7° (*p* = 0.05) and the TKA by an average of 2.6° (*p* = 0.03) compared to immediate post-operative measurements. However, anterior and mid-body height measurements remained stable, showing no significant changes (Table 2, Figure 4).

Patient-reported outcomes showed significant improvements. The VAS score for back pain decreased from a preoperative mean of 8.1 ± 1.2 to 1.6 ± 1.4 postoperatively (*p* < 0.01). Similarly, the ODI score improved from 59.7 ± 9.6 to 28.9 ± 12.1 (*p* < 0.01) (Table 3).

**Figure 5:**
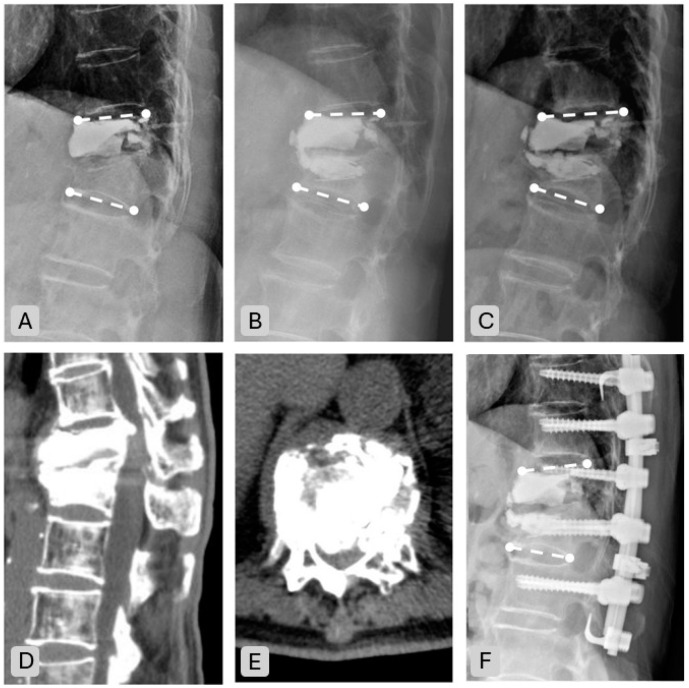
Radiograph and CT images of an 86-year-old female patient with complications post-vertebroplasty. Initially diagnosed with a T12 vertebral compression fracture. (**A**): Lateral X-ray one week after the initial VP at T12 revealed a new adjacent fracture at L1. (**B**): Post-repeated vertebroplasty imaging showed improved kyphotic angle and increased vertebral body height (**C**): Five-month follow-up revealed imaging and clinical deterioration with persistent lower back pain, right thigh numbness, muscle atrophy, and limping gait. (**D**,**E**): CT imaging demonstrated severe spinal cord compression. (**F**): Posterior decompression and instrument fusion was performed to address the severe cord compression.

The overall complication rate was 34.1% (14 cases). Three patients (7.3%) developed nonunion, and de novo fractures occurred in four patients (9.8%)—three in adjacent segments (7.3%) and one in a distant segment (2.4%). Cement leakage was observed in four patients (9.8%, two disc and two paravertebral leakage). Spinal canal and foraminal compromise occurred in three patients (7.3%), resulting in neurological deficits in two patients and persistent back pain in one patient. These complications led to surgical revision in three cases (7.3%) (Table 4, Figure 5).

## 4. Discussion

The utilization of VP in cases of ASF has been previously documented following lumbar interbody fusion surgeries [19,20]. While many studies have explored the risk factors associated with adjacent fractures following VP [21], the application of repeated VP for treating these fractures has only been sporadically reported within the context of risk factor studies. Consequently, our study represents the first comprehensive report of a series of patients who have undergone repeated VP for the management of ASF.

Previous studies on primary vertebroplasty have reported significant improvements in kyphotic angle following VP, with postoperative restoration of the height of the vertebral body [10,22,23]. Meta-analyses comparing VP to non-operative treatments have consistently reported superior pain control with VP, resulting in improvements in health-related quality of life outcomes within 3 months of surgery [9,24,25].

In our study, we observed a significant improvement of 4.8° immediately after adjacent vertebral fracture vertebroplasty. Additionally, the measurement of the kyphotic angle, encompassing both newly treated and previously fractured segments, also showed enhancement after repeated VP, albeit to a lesser extent of 3.0°. The results of our study suggest that repeated VP is comparable to primary VP in terms of postoperative reduction in kyphotic angle [22]. However, our findings indicated a diminished improvement in the kyphosis angle at the final follow-up, although it still represented an improvement compared to pre-surgery measurements. This reduced improvement could be attributed to natural degeneration and the progression of the fracture during the healing period. Repeated VP was also associated with a significant increase, both 3mm in the anterior and middle vertebral body, which is comparable with the results reported in studies of primary VP (2.5–5.1 mm anteriorly, 2.7–4.7 mm centrally) [26]. We believe that the combination of improved sagittal alignments and the stabilization of vertebral fractures contributed to the enhancement of patient-reported outcomes [27]. Notably, the decrease in the mean VAS score and ODI scores both achieved minimal clinically important difference (MCID), indicating a significant improvement in patient-reported outcomes [28].

While vertebral augmentation procedures have demonstrated their effectiveness, concerns regarding potential complications have been raised. In our study, we observed an overall complication rate of 34.1%. It’s important to note that VP complications are typically categorized as mild, moderate, and severe. [29] Mild complications, such as cement leakage into disc and paravertebral soft tissues, are a frequently reported issue in VP procedures, with rates ranging from 5.0% to 87.0% in existing literature [23,30,31,32,33]. In our study, cement leakage occurred in 9.8% of patients, and all cases were clinically asymptomatic. De novo fractures were also classified as a mild complication, and our findings post-repeated VP are comparable with previous studies on primary VP, where the incidence ranged between 2.5% and 18% [32,33,34]. Moderate complications in VP include cement leakage into the epidural/foraminal space and needle misplacement. Our study found that 7.3% of patients who underwent repeated VP experienced epidural/foraminal space cement leakage, leading to reoperation. Review articles have noted that many such cases remain clinically undetected unless post-procedure computed tomography (CT) scans are performed, with prevalence potentially as high as 40% [29,35]. The rate of radiculopathy after primary VP ranged between 3.0–3.9% with an incidence rate of revision surgery at 1.4% [16,23,36], both of which are lower than the rate found in our study for repeated VP. Thus, while repeated VP is effective in treating ASFs, its associated complications should be carefully considered.

The decision to perform repeated VP requires careful consideration of risk-benefit trade-offs, particularly in elderly patients with comorbidities. For elderly patients with multiple comorbidities, the minimally invasive nature of repeated VP may still present a more favorable risk profile compared to open surgical alternatives, particularly given the documented pain relief and functional improvement. Nevertheless, the increased risk of complications compared to primary VP necessitates thorough patient counseling and careful case selection. Factors such as bone quality, existing comorbidities, and the patient’s functional status should be carefully evaluated when considering repeated VP.

The study has several limitations, including its retrospective design and the limited number of cases due to the niche nature of the disease and treatment method. Additionally, the lack of a control group may limit the generalizability and ability to draw firm conclusions about the efficacy of repeated VP for ASF. Despite these limitations, our study is one of the few that discusses the outcome of repeated VP on adjacent fractures, showing the potential effectiveness of this method along with the increased risk of side effects. Future research with larger sample sizes and randomized controlled designs is needed to further investigate the efficacy and safety of repeated VP for ASF.

## 5. Conclusions

Repeated VP for ASF can effectively improve vertebral alignment and patient-reported outcomes in appropriately selected patients. Given the procedure’s risk profile, future research with larger sample sizes and randomized controlled designs is needed to better define optimal patient selection criteria and refine surgical techniques to minimize complications.

## Figures and Tables

**Table 1:** Study Population Demographics.

Characteristic	Value
Age (years)	80.0 ± 7.2
Sex (Men/Women)	4:37
Body mass index (kg/m^2^)	23.9 ± 5.3
Bone mineral density (gm/cm^2^)	−2.6 ± 1.2
Hounsfield unit of vertebral body (HU)	27.0 ± 37.0
Follow-up time (month)	20.1 ± 11.5
Comorbidities, n (%)	
Cardiovascular disease	15 (36.6)
Diabetes Mellitus	5 (12.2)
Chronic kidney disease	6 (14.6)
End-Stage Renal Disease	1 (2.4)
Rheumatic diseases *	2 (4.9)
Steroid use	10 (24.4)
Parkinsonism	2 (4.9)
Cancer	3 (7.3)
Smoking, n (%)	2 (4.9)
Alcohol use, n (%)	0 (0)
The position of AVF, n (%)	
Superior to previous fracture	22 (53.7)
Inferior to previous fracture	18 (43.9)
Sandwich vertebral fracture ^†^	2 (4.9)

Continuous variables were presented as mean ± SD; * rheumatic diseases include systemic lupus erythematosus and rheumatoid arthritis; ^†^ sandwich vertebral fractures: a fractured vertebral body located between two previously fractured vertebrae; one patient suffered from adjacent fractures located superior and inferior of the initial fracture.

**Table 2:** Radiographic Outcomes.

Parameters	Preoperative	Postoperative	Final	* *p* Value	^†^ *p* Value
Kyphotic deformity (°)					
Single kyphotic angle ^‡^	13.4 ± 9.7	8.6 ± 7.0	10.3 ± 9.1	<0.01	0.05
Two-segment kyphotic angle ^§^	15.6 ±11.8	12.6 ± 10.0	15.2 ± 10.1	0.02	0.03
Vertebral body height (cm)					
Anterior vertebral body height	1.9 ± 0.7	2.2 ± 0.6	2.3 ± 0.6	<0.01	0.2
Mid vertebral body height	1.7 ± 0.5	1.9 ± 0.5	1.9 ± 0.5	<0.01	0.8

* Comparison between preoperative and postoperative measurements; ^†^ comparison between postoperative and final follow-up measurements; ^‡^ single kyphotic angle represents the sum of kyphotic angles in all segments receiving repeated VP; ^§^ two-segment kyphotic angle represents the combined kyphotic angle of previous and new VP segments.

**Table 3:** Patient Reported Outcomes.

Outcome Measure	Preoperative	Postoperative	*p* Value
VAS pain score *	8.1 ± 1.2	1.6 ± 1.4	<0.01
ODI score (%) ^†^	59.7 ± 9.6	28.9 ± 12.1	<0.01

* VAS: Visual analog scale (0–10, higher scores indicate worse pain); ^†^ ODI: Oswestry Disability Index (0–100%, higher scores indicate greater disability).

**Table 4:** Complications Following Repeated Vertebroplasty (n = 14).

Complication Type	n (%)
Nonunion	3 (7.3)
New onset fracture	4 (9.8)
Adjacent	3 (7.3)
Remote	1 (2.4)
Cement leakage	4 (9.8)
Disc leakage	2 (4.9)
Vessel leakage	2 (4.9)
New neurologic deficits	3 (7.3)
Spinal stenosis	2 (4.9)
Persistent back pain	1 (2.4)
Re-operation	3 (7.3)

## Data Availability

Study data are available upon request.
